# Remedy for Infantile Asphyxia

**Published:** 1853-05

**Authors:** W. P. Jones


From the Southern Journal of the Medical and Physical Sciences.

*Remedy for Infantile Asphyxia.* By W. P. JONES.

ONE pleasant afternoon, not long ago, I was sitting quietly in my office, when my cogitations were interrupted by the hasty steps of a large, fresh, Yankee looking gentleman, who said his wife was suffering very much and wanted a Doctor. I followed him, and on the way, was informed by the messenger that he was the husband of the sufferer, and had only been married a few days—an interesting patient thought I, and probably quickened my steps. On arriving at the house, I was conducted into the room, where I soon became apprehensive that the woman, though just married, was absolutely in child birth, and that my services as accoucher, would be brought into immediate requisition. After making the usual examination, and becoming thoroughly satisfied that my conjecture was well founded, and that it was the first presentation, I sat down to await the issue, and was not long detained; for immediately very severe pains supervened, and a fœtus was expelled. Without, however, any other vital indication than bodily warmth, and very feeble, almost indistinct, circulation in the cord.

The ordinary means, such as stimulating baths, inflating the lungs, friction, &c., were used, probably a half hour or more, without any apparently beneficial effect. At this crisis, the father, (or man who should have been,) entered the room, bearing upon his breath the odor of alcohol. I called to him and told him to come and apply his lips to those of the child, and gently blow into its mouth. He looked astonished, and exclaimed: What, me, sir!! Yes sir, said I, you are just the man, come on. He did so. He inflated the lungs and I expelled the air repeatedly; and together we at length succeeded in resuscitating the child, which I am constrained to regard as wholly attributable to the stimulus of the alcoholic inspiration. It is at any rate worthy of a trial, after the ordinary means have failed; and in the absence of a drunken husband, we suggest that the accoucher inhale the vapor of alcohol or spirits or camphor, immediately previous to inflating the lungs of the child.

